# Development of Pharmaceutical Nanomedicines: From the Bench to the Market

**DOI:** 10.3390/pharmaceutics14010106

**Published:** 2022-01-03

**Authors:** Abdulrahman A. Halwani

**Affiliations:** 1 Pharmaceutics Department, Faculty of Pharmacy, King Abdulaziz University, Jeddah 22252, Saudi Arabia; aahalwani@kau.edu.sa; 2 Regenerative Medicine Unit, King Fahd Medical Research Center, King Abdulaziz University, Jeddah 22252, Saudi Arabia

**Keywords:** nanotechnology, pharmaceutical nanomedicines, nanoparticles, drug delivery

## Abstract

Nanotechnology plays a significant role in the field of medicine and in drug delivery, mainly due to the major limitations affecting the conventional pharmaceutical agents, and older formulations and delivery systems. The effect of nanotechnology on healthcare is already being felt, as various nanotechnology applications have been developed, and several nanotechnology-based medicines are now on the market. Across many parts of the world, nanotechnology draws increasing investment from public authorities and the private sector. Most conventional drug-delivery systems (CDDSs) have an immediate, high drug release after administration, leading to increased administration frequency. Thus, many studies have been carried out worldwide focusing on the development of pharmaceutical nanomedicines for translation into products manufactured by local pharmaceutical companies. Pharmaceutical nanomedicine products are projected to play a major role in the global pharmaceutical market and healthcare system. Our objectives were to examine the nanomedicines approved by the Food and Drug Administration (FDA) and the European Medicines Agency (EMA) in the global market, to briefly cover the challenges faced during their development, and to look at future perspectives. Additionally, the importance of nanotechnology in developing pharmaceutical products, the ideal properties of nanocarriers, the reasons behind the failure of some nanomedicines, and the important considerations in the development of nanomedicines will be discussed in brief.

## 1. Introduction

Nanotechnology and nanosciences are commonly seen as providing a significant advantage to many areas of study and applications [1]. Nanotechnology means the production and use of materials, equipment, and systems in nanoscale, which is an intermediate range between atoms and the molecular scale with the important prerequisite that at least one dimension is in the nanometer length [2,3]. The effect of nanotechnology on healthcare is already being felt, as different nanotechnology ideas have been developed, and several nanotechnology-based medicines are now available in the market. Across many parts of the world, nanotechnology draws expanded investments from public authorities and the private sector [2].

Many studies have been undertaken to discover and improve new medicines that have the ability to target disease sites much more precisely. Nanotechnology allows therapeutic agents to reach the target site of disease by overcoming their limitations [4]. Many drugs have limitations when it comes to the route of administration due to issues surrounding solubility, permeability, and bioavailability, leading to poor pharmacokinetics. The goal is thus to develop a dosage form with a proper pharmacokinetics delivery system. Nanoparticle-based drugs can either be therapeutic agents themselves or act as a carrier for transporting different therapeutic agents to certain parts of the body.

Nanoparticle-based drugs display a promising approach to obtaining desirable drug-specific properties by manipulating the biopharmaceutical and pharmacokinetical properties of the molecule. Reducing the undesirable toxicity from nonspecific distribution, improving patient adherence, and providing beneficial clinical findings are the main advantages of using nanotechnology in improving disease targeting by therapeutic agents using nanotechnology to deliver such agents with indirect minimizing of the burden on the healthcare system. In general, the development of new medicines and therapies that are focused on nanotechnology is driven by the need to build therapies that are less toxic and cheaper than conventional treatments.

Since 1989, the number of approved nano-based pharmaceutical applications and products has significantly increased [1]. Over the last 20 years, around 80 nanomedicine products have been approved by the Food and Drug Administration (FDA) and the European Medicines Agency (EMA) for marketing (Table 1). This shows the importance of nanotechnology in the field of drug delivery. Several nanomedicines have been used to increase effectiveness and reduce adverse reactions through changes in the efficacy, safety, and physicochemical and pharmacokinetic/pharmacodynamic characteristics of the original medicines [5].

In this review, we discuss the importance of nanotechnology in developing pharmaceutical products, the ideal properties of nanocarriers, the reasons behind the failure of some nanomedicines, and the important considerations in the development of nanomedicines. Our objectives were to sum up the different approved nanomedicines on the global market, to briefly cover the challenges faced during the development of nanomedicines, and to look at the future prospects.

## 2. The Importance of Nanotechnology in Developing Pharmaceutical Medicines

Nanotechnology plays a significant role in the field of medicine and in drug delivery, mainly due to the major limitations and problems affecting conventional pharmaceutical agents, and older formulations and delivery systems [1]. Poor delivery to the target site is one of the inefficiencies of certain currently available medicines [3]. For this reason, drug delivery in therapeutics is a significant factor. Most conventional drug-delivery systems (CDDSs) have an immediate, high drug release after administration, leading to increased administration frequency [1]. Misuse is one of the drawbacks associated with increased administration frequency that can lead to drug toxicity. It is also a major challenge for pharmaceutical companies in developing new medicines, as drug solubility in the CDDSs tends to be low, hence affecting efficacy [1,3]. Moreover, low drug stability is one of the major limitations of using conventional pharmaceutical agents, as the CDDSs in some dosage forms are not sufficient to protect the active pharmaceutical ingredients (APIs) against biological fluids in the body. Accordingly, solving all previous issues related to drug delivery would help in improving quality of life and the healthcare system, and in reducing healthcare costs. A reduction in overall healthcare costs is one of the beneficial features of nanomedicines. There are pressures in many countries to reduce overall healthcare costs. Thus, many studies have been carried out worldwide focusing on the development of pharmaceutical nanomedicines, translating to products being manufactured by local pharmaceutical companies.

The above-mentioned issues related to CDDSs can be successfully resolved by applying nanotechnology to the development of pharmaceutical medicines. The use of nanoparticles (NPs) in drug production has multiple benefits compared with the use of CDDSs, such as: (1) delivering therapeutic agents more specifically to the targeted tissue and reducing total dose and potential toxic side effects; (2) improving the stability of the APIs after administration and thus their bioavailability; (3) demonstrating better safety and efficacy; (4) releasing drugs at a constant rate over a desired timescale; (5) allowing passive targeting and the accumulation of drugs in malignant tumors and other pathological sites through the enhanced permeability and retention (EPR) effect; and (6) nanopharmaceutical products can be far cheaper than conventional ones [1].

## 3. Ideal Properties of Nanoparticle Delivery Systems

Nanoparticles are colloidal particles, ranging from 1 to 1000 nm, and are mainly composed of different macromolecules in which the therapeutic drugs can be adsorbed, entrapped, or covalently attached. In the field of drug development, nanoparticles provide significant advantages that can be attributed to their physicochemical properties in order to achieve controlled release characteristics [6]. Controlling the product life cycle through reducing dosing frequency creates a potential opportunity that can be achieved by designing nanoparticles with controlled release for drugs that go off-patent. Numerous factors impact the effectiveness of nanoparticles in drug delivery, such as physical and biological stability, good component tolerability, the simplicity of the manufacturing method, ease of manufacturing process scale-up, the convenience of freeze-drying, and sterilization [6]. There are some properties of nanoparticles that are important for drug-delivery application and should be taken into consideration during the development of nanomedicines:

1. Nanoparticles should be characterized by high drug capacity and exhibit a very low possibility of immediate release of the therapeutic agent.
2. Nanoparticles should have the ability to be combined with ligands for targeted drug delivery.
3. Nanoparticles should be stable enough to pass through the biological barriers with respect to their physicochemical properties, such as size, size distribution, zeta potential, etc.
4. Therapeutic agents should be completely released from nanoparticles at an optimal rate that is based on the formulation design.
5. Nanoparticles should be biocompatible, biodegradable, and nonimmunogenic.
6. Organic solvents and toxic ingredients should be excluded from the manufacturing process.
7. All components of the formulation should be safe, affordable and commercially available.
8. Simplicity, affordability, and ease of scaling up are the main characteristics that should be included in the manufacturing process.
9. Nanoparticle formulation should have the ability to be involved in different processes during the manufacturing process, such as lyophilization, sterilization, drying, blending, granulation, compression, capsule filling, and packaging.
10. Nanoparticles should be stable in storage.

Biocompatibility, biodegradability, and nonimmunogenicity are the most essential characteristics that should be applied to nanoparticle delivery systems. These characteristics are being studied extensively for nanocarriers to have efficient drug delivery with improved bioavailability and reduced side effects. To ensure high biosafety for in vivo applications, an ideal drug nanocarrier should have high biocompatibility and good biodegradability. Many inorganic drug nanocarriers, such as metals, metal oxides, and carbon-based materials, are difficult to degrade, which can result in toxicity and limit clinical applications. Lipid-based nanoparticles and polymer-based nanoparticles are the most useful nanocarriers for drug delivery in most global approved nanomedicines (Table 1), due to their biocompatibility, lack of intrinsic toxicity, and improved drug-loading capacity. In addition, these nanocarriers may provide special protection for therapeutic agents against environmental degradation. The surfaces of liposomes and micelles can also be functionalized to improve pharmacokinetic profiles of therapeutic agents.

## 4. Challenges in the Development of Pharmaceutical Nanomedicines

The field of pharmaceutical nanotechnology has seen enormous growth and progress over the last 20 years. Particular attention has been paid to the field of nanomedicine, as it promised to revolutionize medical care through more efficient, less toxic, and more intelligent therapies that can be targeted to the site of disease [7]. Many nanomedicines have been developed successfully and approved for clinical use, with significant effort from both academia and the biopharmaceutical industries [1,8]. However, the field of nanomedicine is still in its infancy with few success stories, as various types of challenges are faced during the development of pharmaceutical nanomedicines. These challenges can be categorized as follows.

### 4.1. Challenges in Drug Delivery across Different Biological Barriers

Drug-loaded nanoparticles have been developed for different routes of administration, such as nasal, oral, transdermal, ocular, and parenteral [9,10,11,12,13]. There are several biological barriers that prevent NPs from reaching their desired disease sites successfully, which mainly depends on the targeted organ and the route of administration [7,14]. Oral administration is the most preferred route for drugs and bioactive molecules. However, the low solubility, stability, and bioavailability of many drugs are the main challenges for oral administration [15]. It is difficult to deliver some drugs through the gastrointestinal tract (GIT), as they become inactive in the GIT before being absorbed, mainly due to the harsh conditions in the GIT system, including the wide range of pH across the GIT and the presence of large numbers of degrading enzymes. Furthermore, the mucus layers that cover epithelial surfaces are significant barriers to the penetration of NPs through the gut epithelium, as the rapid secretion and shedding of GIT mucus can significantly limit the effectiveness of nanoparticulate drug-delivery systems [16,17].

Generally, the local delivery of therapeutic agents has fewer barriers than systemic delivery. The systemic delivery of therapeutic agents, especially genes, proteins, and peptides, faces significant challenges in traveling from the site of administration to the site of action [14]. Briefly, after therapeutic agents reach the bloodstream following intravenous administration, they travel through the circulatory system, and hence accumulate in nontargeted organs of the reticuloendothelial system (RES), including the liver, lungs, and spleen. In general, biotherapeutic agents aggregate with proteins in the serum, are degraded by endogenous enzymes, and are taken up by nontargeted cells such as phagocytes [14,18,19]. This increases the systemic elimination of biotherapeutic agents, resulting in a short plasma half-life after intravenous administration [14].

One of the most essential defense mechanisms in the central nervous system (CNS) is the blood–brain barrier (BBB). Drug movement through the BBB is mainly limited by the occurrence of tight junctions, rather than large fenestrations, between endothelial cells [20]. Only small molecules, including water, gases, and other lipid-soluble compounds, can penetrate passively through the BBB. Conversely, transports of large molecules with high electric charges, polarity, and hydrophilicity, such as glucose, amino acids, and most drugs by active routes of transportation, will rely on specific proteins [21]. The delivery and release of therapeutic agents into the brain is a challenging area. Therefore, NPs that have been developed and applied to cross the BBB mainly include polymeric NPs, such as poly(butylcyanoacrylate) (PBCA) [22], poly(lactic-co-glycolic acid) (PLGA) and poly(lactic acid) (PLA) NPs [23], liposomes [24], and inorganic composites such as gold, silver, and zinc oxide NPs [25,26,27].

Another critical challenge is addressing the difficulty of delivering therapeutic drugs into solid tumors, as wide areas of tumors may not be well permeated. Tumor vasculature in some cases is highly heterogeneous in distribution and more permeable, but large tumor areas may have little perfusion [28,29]. The poor perfusion of blood in solid tumors can be explained by five major abnormal physical and physiological properties: (a) a solid tumor mass compresses the blood vessels, resulting in a minimization of the drug supply to several tumor regions; (b) immature vasculature with high viscous and geometric resistances and low pressure gradients allow the blood in a tumor to slow down and heterogeneously limit drug supply; (c) high metabolic consumption rate of glucose resulted in significant production of lactate and H^+^ that leads to low pH media, (d) nonfunctional lymphatics and highly permeable blood vessels cause high hydrostatic pressure in tumors, stopping the continuous movement of drugs from blood vessels into tumor tissue; and (e) the dense structure of the cells and the interstitial matrix acts as a steric barrier for the diffusion of therapeutic agents [30]. Therefore, the essential pharmacokinetic steps that must be performed for the delivery of drugs into tumor cells include transport and metabolism in cells, vascular transport, and interstitial transport [30]. For example, the high concentration of H^+^ in the tumor can diffuse into adjacent cells, resulting in an acidic microenvironment for neighboring normal cells. Accordingly, some nanomedicines have been developed recently to target the pH of tumors through activation of prodrug, or drug release from nanocarriers by low pH of the tumor or using drugs that raise the pH of the acidic tumor [31].

### 4.2. Challenges in the Formulation, Characterization, and Manufacturing of Nanomedicines

Nanomedicines are likely to be three-dimensional constructs with multiple components in ideal spatial arrangements. Therefore, slight changes in the method or composition of these constructs can influence the dynamic superposition of the components [7]. Highly reproducible manufacturing processes for nanomedicines can be achieved by understanding the physicochemical characterization of the components, understanding the critical components and their interactions, and identifying the main features and their performance relationships [7].

#### 4.2.1. Physicochemical Characterization of Nanoparticle Components

The ways nanoparticles act in vitro and in vivo depend on several physicochemical characteristics, including size and size distribution, surface morphology, surface chemistry, surface charge, surface adhesion, steric stabilization, drug-loading efficiency, drug release kinetics, and the hemodynamic properties of the nanoparticles [7]. Nanoparticles have been developed and adapted to deliver different kinds of therapeutic agents, including small-molecule drugs, peptides, proteins, nucleic acids, and genes. Liposomes, polymers, proteins, micelles, dendrimers, quantum points, nanoshells, nanocrystals, gold nanoparticles, paramagnetic nanoparticles, and carbon nanotubes are all nanoparticles used in drug delivery [32]. Each system varies greatly in its architecture and has special key characteristics that may help to improve the performance of nanoparticles in particular indications. However, it is crucial to identify the appropriate nanoparticle parameters for particular indications. Going one way may fix a specific problem, but may also lead to another [7]. Doxil^®^ is a good example to illustrate how the physicochemical properties of nanoparticles can affect pharmacokinetics. Non-PEGylated liposomes, containing doxorubicin, are extremely affinous to the RES and are easily removed from circulation due to a process called “opsonization”, while PEGylated liposomes can minimize their affinity to the RES and hence decrease the uptake of liposomes by macrophages significantly. Doxil^®^ is a PEGylated liposomal doxorubicin that shows a long half-life, an increased concentration of tumor drugs, an improvement in antitumor effectiveness, and fewer side effects than non-PEGylated ones.

#### 4.2.2. Analysis and Characterization of Nanoparticle Formulation

The identification of proper analytical tests to complete the characterization of nanoparticles may be one of the most challenging areas of nanomedicine development. As nanomedicines have a complex nature compared with conventional pharmaceutical products, and each component of nanomedicines has a specific function, a more advanced testing level should be applied to fully characterize nanoparticles to ensure that they have all the desired properties for the intended therapeutic purpose [7]. Several proposed nanomedicines have biological components, such as proteins or nucleic acids [33,34], that may be sensitive to conditions in the manufacturing process and, hence, compositional changes may occur from processing in some cases [7]. These biological components may not be the active pharmaceutical ingredients in nanomedicines, but they may have a role to play in targeting certain cells or in distributing the active components in the body. Thus, suitable analytical tests should be applied to these components in order to obtain a full characterization, and they cannot be regarded as an inactive excipient due to their important role in the effectiveness or the protection of the drug [7]. The better the understanding of the components of nanomedicines in the early stages of development, the more likely an effective reproducible manufacturing process will be achieved [7].

#### 4.2.3. Scale-Up and Manufacturing

In terms of pharmaceutical development, the successful scale-up and production of nanomedicines presents difficulties and challenges. Traditional drug production does not normally create nanometer-scale, three-dimensional multicomponent systems, a fact that presents several barriers to the scale-up of nanomedicines. As most pharmaceutical nanoparticles are complex in nature with multiple components in ideal spatial arrangements, a thorough understanding of the critical components and their interactions is important for identifying the key characteristics of the product. The early identification of these characteristics in the development of nanomedicines will assist in the choice of a suitable large-scale manufacturing method to establish the critical process steps and analytical criteria that will ensure the reproducibility of the product [7].

The formulation of nanoparticles usually requires the use of organic solvents, sonication, high-speed homogenization, milling, emulsification, crosslinking, evaporation of organic solvents, filtration, centrifugation, and lyophilization. At an early stage of development in the laboratory, it is helpful to consider, on a “small scale”, which method may be most beneficial for the scaling-up of the product [7]. A sterile process of manufacturing nanomedicines that uses a special route of administration will face challenges, depending on the size and composition of the particles. One of these challenges is that the risk of nanoparticles being damaged by sterilization techniques such as gamma irradiation or autoclaving will increase, especially when biological materials are involved [35,36,37]. The sterilization of nanoparticles through conventional sterile filters may not be an issue if the structure of the particles is flexible, as with liposomes, especially if their particle size is well below 220 nm. For hard-structure nanoparticles, such as polymeric, metal, silica, and others, sterilization through filters may be the only choice. However, large particle-size distribution and a particle size closer to 220 nm may result in great filtration difficulties due to the small pore size of standard filtration membranes. Accordingly, enormous amounts of active ingredients could be lost on filtration if the average particle size is not well below 220 nm [7]. Although aseptic manufacturing is often a suitable choice, it can be quite complicated, especially for a multistage process involving handling and the transfer of materials in a sterile environment.

Environmental safety is a further problem for the manufacturing of nanoparticles. Nanoparticles can spread in the air during the handling of dry materials, which leads to deposition of the nanoparticles in the lung causing pulmonary toxicity [38,39]. Therefore, extreme caution is required during the manufacturing of nanomedicines. In addition, sufficient personal protective equipment is essential during manufacturing, as some nanoparticles are able to penetrate the skin barrier, making dermal exposure a potential risk [38]. Nanoparticles that are manufactured entirely in the liquid environment, mostly similar to the standard manufacturing of pharmaceutical liquids, may have a significantly lower environmental impact.

#### 4.2.4. Challenges in the Regulation of Nanomedicine Development

In comparison to conventional pharmaceutical products, in which a single active agent is normally used, most pharmaceutical nanoparticles are complex in nature, with multiple components and heterogenous structures, where more than one component can affect the pharmacological behavior of the active ingredient. Due to this complexity, the regulation of nanomedicines may face several obstacles [7,40]. Currently, new medicines based on nanoparticles are evaluated by the Food and Drug Administration (FDA), the European Medicines Agency (EMA), and other agencies using a case-by-case approach under the traditional framework of benefit/risk analysis [7,41,42]. In general, there is a lack of standards in the evaluation of nanomedicines, as they are a unique category of therapeutic agents [7].

Generally, pharmaceutical products are regulated by the FDA under two main laws: the Federal Food, Drug, and Cosmetic Act (FDCA), which covers all chemically synthesized drugs and devices; and the Public Health Service Act (PHSA), which covers biologically derived therapeutic products [40,41]. The definitions and policies of these laws differ among three product areas based on whether the product includes a chemical action mode (drug), mechanical action mode (device), or biological source [41]. The FDA has not published any particular guidance for nanomedicines until recently, as they are categorized under complex products with multiple components. In other words, the FDA makes no categorical assessment of nanomedicines as being safe or harmful, and will continue to consider the particular characteristics of individual products. In December 2016, provisions for transparency and consistency in FDA procedures were established for classifying and evaluating combination products. A combination product is defined as a product that contains a mix of three product areas: a drug and a device; a drug and a biologic; a device and a biologic; or all three types of products [41]. Nanomedicines are categorized by the FDA as combination products, assigned by the traditional regulatory route and supplemented with special requirements to assure safety and efficacy. For example, paclitaxel and doxorubicin nanoformulations have been approved by the FDA as new cancer drugs, classified as combination products. Due to the debate on the adequacy of current regulatory frameworks and procedures, wider concerns have been raised about the inherent risks of nanotechnology and products containing nanoparticles, including nanoparticle toxicity, the unintended effects of nanoparticles’ ability to cross the BBB, and the long-term effects of nanoparticles [41,43,44]. Accordingly, the FDA has issued one draft and five final guidance documents discussing the use of nanotechnology in FDA-regulated products, including nanomedicines. All six guidelines encourage manufacturers to consult the company before marketing their products.

Nanomedicines are categorized according to the EMA into biological and nonbiological medicines [45]. More comprehensive study beyond plasma concentration measurement is required for biological and nonbiological nanomedicines. A step-by-step comparison of the nanomedicine with a reference medicine is required, including bioequivalence, safety, quality, and efficacy, which will lead to therapeutic equivalence [42,46]. The biological framework can sometimes be considered as the basis for the regulation of nonbiological complex drugs (NBCDs), as there are certain features in common: the structure is not fully characterized, and the in vivo activity is dependent on the process of manufacture, which means that comparability needs to be made throughout the life cycle, as with biological nanomedicines [42,47]. In the field of nanomedicine, the EMA has already published several reflection papers. These papers are used to provide advice to developers in the processing of marketing authorization applications for new nanomedicines and nanosimilars [42].

As we have recently entered the era of generic nanomedicines, both generic drug manufacturers and drug regulators face real challenges in defining a framework for the evaluation of generic nanomedicines to demonstrate that they are bioequivalent to the branded ones and have the same physicochemical properties, as well as being safe and effective [7,42,48]. The connections between the physicochemical properties of nanoparticles and their clinical pharmacokinetics (PK) and safety are poorly understood, and traditional animal models may not be adequate for the proper extrapolation and prediction of the biodistribution and toxicity of nanoparticles in humans. This is particularly important when a novel drug based on nanoparticles is compared with conventional formulations, and when a generic approved nanomedicine is evaluated against a product of innovation. The similar outcomes in general PK and toxicity studies, or a simple comparison of the composition of drug products, cannot be used to assume the bioequivalence of generic and innovator nanomedicines.

## 5. Considerations in Nanomedicine Development

### 5.1. Chemistry, Manufacturing, and Controls (CMC) Considerations

Nanomedicines are more complex than conventional drugs, as they face CMC challenges during product development and in the later stage of manufacturing scale-up. Thus, the first step in developing nanomedicine is to determine practicability through understanding the makeup and structure of the early formulation to prove the principle in the research. This will ensure that the formulation can be reproducible during confirmatory studies, as well as ensure its future safety and efficacy in clinical trials [4]. It is essential that the early nanomedicine candidates have adequate physical, chemical, and functional characterization. Analytical techniques such as nuclear magnetic resonance (NMR), mass spectrometry (MS), chromatography, etc., must be used to identify the chemical structure of each component involved in nanoparticle formulation [4]. The physicochemical properties of the early formulated sample; i.e., particle size, zeta potential, pH, viscosity, and purity, also need to be established and understood. The biological functions of nanoparticles are required to be characterized and investigated to provide acceptable levels of confidence.

Moreover, the potential commercial-scale manufacturing of nanomedicine products that are easily reproducible at a reasonable cost is very important. For this reason, the CMC developers should understand the early stages of nanomedicine synthesis and assess whether the chemicals and the processing can be used and performed on an industrial scale. Cytotoxic compounds and complex processing may be achievable on a laboratory scale, but may be expensive and challenging on an industrial scale [4]. The manufacturing process of nanomedicines is quite complex, and so minimizing the batch-to-batch variability of nanomedicine formulations is a major challenge. Thus, the quality of the product, which impacts the strength, purity, safety, and efficacy of nanomedicines, is essential for successful development and commercialization. Being aware of the quality of the starting materials and understanding the processing conditions for preparing nanomedicine formulations allow the developer to control the manufacturing successfully on an industrial scale [4].

### 5.2. Economic Considerations

The amount of investment required to fund the development and scaling-up of nanomedicine production needs to be considered. The overall risk of CMC development will need to be factored into the investment profiles used for analyzing potential nanomedicine programs against other development portfolios. Instrumentations, manufacturing equipment, and other facilities may be costed for some companies, therefore these facilities need to be involved in the investment strategies of nanomedicine development aligned with clinical requirements [4].

### 5.3. Regulatory Considerations

Early FDA consultations regarding nanomedicine development will help in understanding the scientific and regulatory issues relevant to the product, and in addressing questions concerning the safety, effectiveness, public health impact, and regulatory status of the product [4]. Therefore, nanomedicine development should follow the usual pathways and processes of drug development using a suitable evaluation framework. In Europe, the EMA has established an Expert Working Group, and has released some reflection papers for particular nanomedicines in order to provide guidance to developers in the preparation of marketing authorization applications [4,42]. However, it is not clear whether the existing regulatory frameworks will pose challenges in the future for more innovative nanotechnology.

## 6. Developmental Nanomedicines

Pharmaceutical nanomedicine products are projected to play a major role in the global pharmaceutical market and healthcare system. Since 1995, around 70 nanomedicine products have been approved by the Food and Drug Administration (FDA) and the European Medicines Agency (EMA) for marketing [1,5,8,49,50,51,52] (Table 1), and double this number are available in clinical trials. Many reports demonstrate that the number of medications based on nanotechnology are increasing annually. Every year, new nanomedicines of previously approved drugs enter clinical trials to investigate their improvement with regard to efficacy compared with conventional formulations [53]. This is due to the rapid growth in research and development (R&D) and the high market demand, which shows the importance of nanotechnology in the field of drug delivery. However, the majority of nanomedicines approved to date have shown a reduced toxicity rather than improved efficacy [53]. A variety of nanoparticle-based drugs have entered the market and are used daily by many patients (Table 1). These products are from various companies worldwide, and indicate the success of nanomedicines as therapeutic agents.

Since 1989, 78 nanomedicines have been approved and have entered the global market. Of these nanomedicines, 66 have been approved by the FDA, and 31 have been approved by the EMA. Both the FDA and the EMA have shared in the approval of 20 nanomedicines globally, while other nanomedicines have an approval from one side (FDA: 43 nanomedicines; EMA: 12 nanomedicines). Since 2010, focus on the development of nanomedicines and the number of marketed nanomedicines have significantly increased due to the resulting healthcare system benefits. The globally marketed nanomedicines can be classified as nanocrystals, lipid-based nanoparticles, polymer-based nanoparticles, dendrimer-based nanoparticles, protein-based nanoparticles, or inorganic nanoparticles (Table 1).

**Table 1:** List of globally marketed nanomedicines approved by the FDA and the EMA *.

Type	Trade Name	Company	Date of Approval	Active Ingredients	Indication
Nanocrystals	Emend^®^	Merk & Co. Inc.	FDA (2003)	aprepitant	antiemetic drug [54,55]
	Ivemend^®^	Merk & Co. Inc.	FDA, EMA (2008)	fosaprepitant dimeglumine (prodrug of aprepitant)	antiemetic drug [56]
	Ostim^®^	Osartis GmbH & Co.	FDA (2004)	calcium hydroxyapatite	bone-grafting material [57]
	Rapamune^®^	Wyeth Pharmaceuticals Inc. (a subsidiary of Pfizer Inc.)	EMA (2001), FDA (2010)	sirolimus (rapamycin)	prevents rejection of kidney transplants [58] (immunosuppressant)
	Rapamune^®^	Wyeth Pharmaceuticals Inc. (a subsidiary of Pfizer Inc.)	FDA (2015)	sirolimus (rapamycin)	a rare progressive lung disease [58] (lymphangioleiomyomatosis)
	Vitoss^®^	Orthovita Inc.	FDA (2003)	β-tricalcium phosphate	bone-grafting material [59]
	Ritalin LX^®^	Novartis	FDA (2002)	methylphenidate	attention deficit hyperactivity disorder (ADHD) [60] in children
	Avinza^®^	Pfizer Pharmaceuticals	FDA (2002)	morphine sulfate	psychostimulant [61]
	Focalin XR^®^	Novartis	FDA (2008)	dexamethylphenidate HCl	ADHD in children [62]
	Invega^®^	Janssen Pharmaceuticals	FDA (2009)	paliperidone	schizophrenia [63]
	Invega Sustenna^®^	Janssen Pharmaceuticals	FDA (2009)	paliperidone Palmitate	schizophrenia [64]
	Megace ES^®^	Par Pharmaceuticals	FDA (2005)	megestrol acetate	antianorexic [65]
	NanOss^®^	RTI Surgical	FDA (2005)	hydroxyapatite	bone substitute [66]
	EquivaBone^®^	Zimmer Biomet	FDA (2009)	hydroxyapatite	bone substitute [67]
	OsSatura^®^	Isotis Othobiologics Inc.	FDA (2003)	hydroxyapatite	bone substitute
	Epaxal^®^	Crucell Berna Biotech	EMA (1993)	inactivated hepatitis A virus vaccine	prevents hepatitis A infection [68]
	Zanaflex^®^	Acorda	FDA (2002)	tizanidine HCl	muscle relaxant [69]
	Ryanodex^®^	Eagle pharm	FDA (2014)	dantrolene sodium	malignant hyperthermia [70]
	TriCor^®^	Abbott Laboratories	FDA (2004)	fenofibrate	antihyperlipidemia [71]
Lipid-based Nanoparticles	Doxil^®^	Johnson & Johnson	FDA (1995), EMA (1996)	doxorubicin (adriamycin)	metastatic ovarian cancer, HIV-associated Kaposi’s sarcoma (KS) [72]
	Lipodox^®^	Sun Pharma Global FZE	FDA (2013)	doxorubicin hydrochloride	metastatic ovarian cancer, HIV-associated KS [73]
	DaunoXome^®^	Galen Ltd.	FDA, EMA (1996)	daunorubicin	cancers and HIV-associated KS [74]
	Onivyde^®^	Merrimack Pharmaceuticals	FDA (2015)	irinotecan	metastatic pancreatic cancer [75]
	DepoCyt^®^	Pacira Pharmaceuticals	EMA (2002), FDA (2007)	cytarabine	lymphomatous meningitis [76]
	Myocet^®^	Teva Pharmaceutical Industries Ltd.	EMA (2000)	doxorubicin hydrochloride	breast cancer [77,78]
	Caelyx^®^	Janssen Pharmaceuticals	EMA (1996)	doxorubicin	breast cancer, ovarian cancer, HIV-associated KS [79,80]
	Mepact^®^	Takeda France SAS	EMA (2009)	mifamurtide	osteogenic sarcoma [81]
	Marqibo^®^	Talon Therapeutics	FDA (2012)	vincristine	Philadelphia chromosome-negative chronic myelogenous leukemia in adult patients [82,83]
	Onpattro^®^	Alnylam	FDA & EMA (2018)	patisiran	hereditary transthyretin (TTR) mediated amyloidosis [84,85]
	Lipusu^®^		FDA (2016)	paclitaxel	breast cancer, non-small-cell lung cancer (NSCLC) [86]
	AmBisome^®^	NeXstar Pharmaceuticals	EMA (1990), FDA (1997)	amphotericin B	antifungal drug [87]
	Vyxeos^®^	Jazz Pharmaceutics	FDA (2017), EMA (2018)	daunorubicin and cytarabine	acute myeloid leukemia [88]
	Abelcet^®^	Defiante Farmaceutica	FDA (1995)	amphotericin B	antifungal drug [87]
	DepoDur^®^	SkyePharma	FDA (2004), EMA (2006)	liposomal morphine sulphate	postoperative analgesia [89]
	Curosurf^®^	Chiesi	FDA (1999)	poractant alfa	respiratory distress syndrome (RDS) [90,91]
	Zevalin^®^	Bayer Pharma	FDA (2002) Disc. * EMA (2004)	90Y-ibritumomab tiuxetan	lymphoma [92]
	Inflexal^®^	Crucell Berna Biotech	EMA (1997)	inactivated influenza virus vaccine	prevents influenza infection [93,94]
	Pfizer-BioNTech Vaccine	Pfizer Pharmaceuticals	FDA (2020)	mRNA vaccine	prevents COVID-19 infection [95,96]
	Moderna COVID-19 Vaccine	ModernaTX Inc.	FDA (2020)	mRNA vaccine	prevents COVID-19 infection [96,97]
	Visudyne^®^	QLT Phototherapeutics	FDA & EMA (2000)	photosensitizer (PS), benzoporphyrin	choroidal neovascularization caused by wet age-related macular degeneration [87]
Polymer-based Nanoparticles	Cimzia^®^	UCB	FDA (2008), EMA (2009)	IgG Fab’ fragment that specifically recognizes and binds to TNF-α	rheumatoid arthritis [98], Crohn’s disease [99], psoriatic arthritis [100], and ankylosing spondylitis [101]
	Apealea^®^	Oasmia Pharmaceutical AB	EMA (2018)	paclitaxel	ovarian cancer, peritoneal cancer, fallopian tube cancer [102]
	Adagen^®^	Enzon Pharmaceuticals Inc.	FDA (1990)	adenosine deaminase (ADA)	adenosine deaminase (ADA)-severe combined immunodeficiency disorder [103]
	Neulasta^®^	Amgen, Inc.	FDA (2002)	filgrastim	febrile neutropenia, consequent infections arising due to lack of neutrophils [104]
	Oncaspar^®^	Enzon Pharmaceuticals Inc.	FDA (1994), EMA (2016)	L-asparaginase	acute lymphoblastic leukemia, chronic myelogenous leukemia [105]
	Genexol-PM^®^	Lupin Ltd.	FDA (2007)	paclitaxel	breast cancer [106]
	Pegasys^®^	Genentech USA, Inc	FDA, EMA (2002)	recombinant human alfa-2a interferon	hepatitis C [107], hepatitis B [108]
	Diprivan^®^	Fresenius Kabi	FDA (1989), EMA (2001)	propofol	(sedative-hypnotic agent) used in surgeryto induce relaxation before and during general anesthesia
	Somavert^®^	Pfizer Pharmaceuticals	EMA (2002), FDA (2003)	analog of human growth hormone (acts as an antagonist of GH receptors)	acromegaly [109]
	Macugen^®^	Pfizer Pharmaceuticals	FDA (2004)	pegatinib sodium	choroidal neovascularization caused by wet age-related macular degeneration [110]
	Mircera^®^	Vifor	EMA (2007), FDA (2018)	epoetin β (EPO) (EPO is a genetically recombinant form of erythropoietin)	anemia [111]
	PegIntron^®^	Merk & Co. Inc.	EMA (2000), FDA (2001)	alpha interferon (INF) molecule	hepatitis C [112]
	Krystexxa^®^	Savient Pharmaceuticals	FDA (2010)	pegloticase is a recombinant porcinelike uricase	refractory chronic gout [113]
	Plegridy^®^	Biogene	FDA (2014)	recombinant IFN-β	relapsing remitting multiple sclerosis (RRMS) in adult patients [114]
	Adynovate^®^	Baxalta US Inc.	FDA (2015)	coagulation factor VIII	hemophilia A [115]
	Copaxone^®^/FOGA	Teva Pharmaceutical Industries Ltd.	FDA (1996), EMA (2016)	glatiramer acetate	multiple sclerosis (MS) [116,117]
	Eligard^®^	Tolmar Pharmaceuticals Inc.	FDA (2002)	leuprolide acetate	prostate cancer [118]
	Renagel^®^	Sanofi	FDA (2000)	sevelamer carbonate	hyperphosphatemia caused by chronic kidney disease (CKD) [119]
	Renagel^®^/Renvela^®^	Genzyme	EMA (2007)	sevelamer HCL	hyperphosphatemia caused by CKD [120]
	Restasis^®^	Allergan	FDA (2003)	cyclosporine	chronic dry eye [121]
	Rebinyn^®^	NovoNordisk	FDA (2017)	recombinant DNA-derived coagulation FIX	hemophilia B [122,123]
	Estrasorb™	Novavax, Inc.	FDA (2003)	estradiol (17β-estradiol) hemihydrate	moderate vasomotor symptoms due to menopause [124]
	Zilretta^®^	Flexion Therapeutics	FDA (2017)	triamcinolone acetonide	knee osteoarthritis [125]
Dendrimer-based Nanoparticles	VivaGel^®^ BV	Starpharma	FDA (2015)	astodrimer sodium	anti-infective for prevention of recurrent bacterial vaginosis (BV) [126]
Protein-based Nanoparticles	Abraxane^®^	Celgene Pharmaceutical Co. Ltd.	FDA (2005, 2012, 2013), EMA (2008)	paclitaxel	approved by the FDA for treatment of metastatic breast cancer [127] (2005), lung cancer [128] (2012), and metastatic pancreatic adenocarcinoma [129] (2013)
	Ontak^®^	Eisai	FDA (1999)	diphtheria toxin	leukemia, T-cell lymphoma [130,131]
Inorganic Nanoparticles	Feraheme™	AMAG Pharmaceuticals	FDA (2009)	ferumoxytol	anemia [132,133]
	Venofer^®^	Luitpold Pharm	FDA (2000)	iron sucrose	iron deficiency in CKD [134]
	Dexferrum^®^	American Regent	FDA (1996)	iron dextran	iron deficiency in CKD [135]
	Ferinject^®^	Vifor	FDA, EMA (2013)	iron carboxymaltose colloid	iron deficient anemia [136]
	Ferrlecit^®^	Sanofi-Aventis	FDA (1999), EMA (2013)	sodium ferric gluconate	iron deficiency in CKD [137]
	Hensify^®^	Nanobiotix	EMA (2019)	hafnium oxide nanoparticles	locally advanced squamous cell carcinoma [138]
	Infed^®^	Actavis Pharma	FDA (1992)	iron dextran	iron deficiency in CKD [139]
	Feridex^®^/Endorem^®^	AMAG Pharma	FDA (1996) Disc. * 2008 [140]	SPION-dex	imaging agent [141]
	GastroMARK™/Umirem^®^	Mallinckrodt Inc.	FDA (2009)Disc. * 2012 [140]	SPION-silicone	imaging agent [141,142]

* Disc.: discontinued.

Inorganic nanoparticles represent many pharmaceutical carriers that can be used for the cellular delivery of various drugs. Inorganic nanoparticles, such as metal, carbon nanotubes, calcium phosphate, iron oxide, silica, and quantum dot nanoparticles are very attractive for use in drug delivery due to their dual function as diagnostic platforms and as therapeutic carriers [140]. Chemical or biological modifications may be applied to inorganic nanoparticles to meet the ideal requirements for cellular delivery, such as biocompatibility, high charge density, and site-specific delivery [140]. Inorganic nanoparticles exhibit good stability over broad ranges of temperature and pH and are not subject to microbial attack. Despite these obvious benefits, only a small number of inorganic nanoparticle systems have reached the clinic due to slow dissolution and lack of biodegradation, which are the main disadvantages of inorganic nanoparticles, especially for long-term administration [140]. Organic carriers are carbon-based materials that are generally characterized by their biocompatibility, lack of intrinsic toxicity, and improved drug-loading capacity. In these carriers, therapeutic agents are often trapped or bound within the matrix. Organic carriers are mainly classified into three groups, which are lipid-based vectors, polymer-based vectors, and dendrimers. These carriers may provide special protection for therapeutic agents against environmental degradation The surfaces of liposomes and micelles can also be functionalized to improve pharmacokinetic profiles of therapeutic agents.

Cancer is one of the most significant diseases in the world, according to a 2015 WHO factsheet, with 14 million new cases in 2012 and 8.2 million cancer-related deaths [143]. Therefore, new and effective cancer medicines are urgently needed to control the high mortality. Anticancer nanomedicines have made dramatic advancements in the past several decades with the rapid progress of nanotechnologies in medicine. Researchers in academia and pharmaceutical industries have developed several nanoparticles that have the ability to deliver therapeutic and diagnostic agents to tumors selectively to increase the accumulation of chemotherapeutic agents within the tumor [144,145,146]. Different strategies can be used to target the tumor, including: passive targeting based on the enhanced permeability and retention (EPR) effect; active targeting directed by tumor-specific moieties; and stimuli-responsive tumor targeting [147]. Passive targeting is a result of the EPR effect, a phenomenon that leads nanoparticles to accumulate in the tumor tissues due to the leaky nature of the tumor vasculature [148]. Passive EPR targeting and particle-size control were the basic principles of first-generation cancer nanomedicines, such as Doxil, which were simple lipid vesicles shielded with polyethylene glycol (PEG) to avoid opsonization by the cells of the reticuloendothelial system, hence preventing an immune response and prolonging circulation time. Doxil is a pegylated liposomal doxorubicin used to treat different kinds of cancer, including metastatic ovarian cancer and AIDS-related Kaposi’s sarcoma while reducing the drug’s side effects, which can be toxic to various parts of the body, particularly the skin and the heart. Doxorubicin was developed by encapsulating an 80–90 nm size unilamellar liposome coated with PEG [149,150]. Two different mechanisms of doxorubicin have been confirmed to act on cancer cells: intercalation into DNA and the disruption of topoisomerase II-mediated DNA repair, and the generation of free radicals, resulting in damage to cellular membranes, DNA, and proteins [151]. However, doxorubicin has been known to cause severe heart problems, such as heart failure, occurring upon or after treatment [149].

The second strategy for targeting tumors is active targeting, which is based on the interaction between ligand-coated nanoparticles and tumor markers. This strategy of targeting results in an increased accumulation of nanoparticles at the target site or an enhanced cellular uptake of nanoparticles by expressing the target receptor [4]. Abraxane is an example of an active-targeting nanomedicine that was approved by the FDA for the treatment of metastatic breast cancer in 2005, lung cancer in 2012, and advanced pancreatic cancer in 2013. In this product, human albumin is conjugated to paclitaxel in order to deliver it in the form of nanoparticles (130 nanometers in diameter). Biodegradability, lack of toxicity, and immunogenicity make albumin an ideal carrier for drug delivery by causing a potential uptake by cells in tumor and inflamed tissue. The bioavailability of albumin-bound paclitaxel (Abraxane) in the tumor is facilitated by the gp60 receptor (albondin)-mediated pathway in the endothelial cell walls of tumors, which binds to albumin with a high affinity [152,153,154]. The accumulation of Abraxane at the tumor site is also facilitated by SPARC (secreted protein, acidic and rich in cysteine), overexpressed in multiple types of tumors, including breast, prostate, gastric, lung, and kidney. Thus, it should be evident that the response of Abraxane will be enhanced with an increased SPARC level in the tumor [149].

Another innovative strategy for targeting is stimuli-responsive tumor targeting, which focuses on nanoparticles that can be triggered to release their contents on exposure to external stimuli such as heat, light, or ultrasound. ThermoDox is a good example of a stimuli-responsive targeting nanomedicine; it was developed by Celsion Corporation in partnership with Duke University for the treatment of liver cancer. ThermoDox is a temperature-sensitive doxorubicin-pegylated liposome that is able to release the contents of the drug at the target site by elevating the temperature to 39–42 °Celsius via the application of radiofrequency [149]. Moreover, the accumulation of ThermoDox nanoparticles in tumors increases as the blood vessels within the tumors become leaky due to the local application of hyperthermia [149]. Tissue-specific ligand-coated nanoparticles and stimuli-controlled nanoparticle release are the next-generation cancer nanomedicines to target tumors and increase the accumulation of the drug in the tumor.

**Figure 1:**
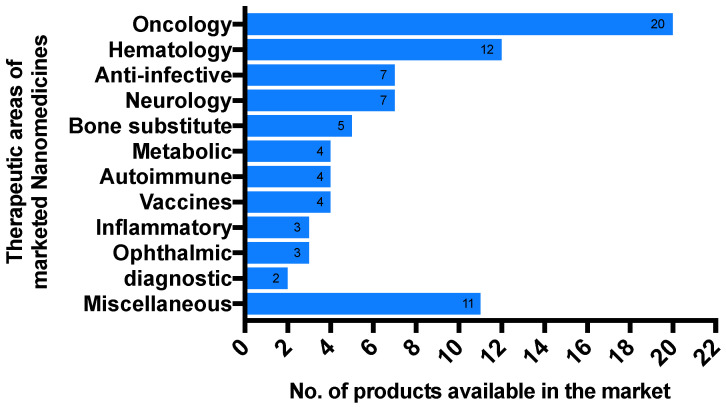
List of globally marketed nanomedicines.

Although more anticancer nanomedicines are currently available on the market than any other drug classes, many formulations are also being marketed for other indications, including autoimmune conditions, metabolic disorders, ophthalmic conditions, neurological diseases, hematological disorders, inflammatory diseases, and others [53] (Figure 1). The majority of the developed nanomedicines have already proven successful use with liposomes and polymers as incorporated NPs [53]. Furthermore, there is a clear trend in the movement of NPs from simple to complex multicomponent drug-delivery systems [154]. These complex systems can involve functions such as controlled release and active targeting to allow nanomedicines to improve PK, efficacy, and safety. While most FDA-approved nanomedicines are based on passive targeting via the EPR effect, some next-generation nanomedicines use active-targeting approaches in clinical trials [4,147,155] (Table 2).

A good example of an active-targeting nanomedicine that has been in clinical trials is MAGE-A3 + AS15, an immunotherapeutic product that is given to patients with carcinomic tumors after the removal of the tumor [158]. Several tumor antigens are encoded by genes of the MAGE-A family that are expressed in various tumor types, such as melanoma [159,160], non-small-cell lung cancer [161], bladder cancer [162,163], and liver cancer [164]. However, these antigens are not expressed in normal tissues, except male germline cells or placenta [165]. Therefore, antigen-based active immunotherapy is an attractive way to fight against cancer, as it has the potential to prepare the immune system of the patient to eliminate the tumor cells and to prolong their lives. Different studies have claimed that the MAGE-A3 protein alone without an immunostimulant has shown limited immunologic and clinical responses [166,167]. A novel immunostimulant, which is a combination of QS21, monophosphoryl lipid A, and CpG7909 (a TLR-9 agonist) in a liposomal formulation (AS15), was associated with a more robust cellular and humoral response in phase II clinical trials compared with other immunostimulants. Therefore, this AS15 immunostimulant has been chosen for further advanced clinical trials [158]. Two phase III clinical trials proceeded with MAGE-A3 + AS15 for melanoma and non-small-cell lung cancer, but both have been terminated, as MAGE-A3 + AS15 was not efficacious and did not increase disease-free survival compared with the placebo (Table 2).

## 7. Potential Causes for the Failure of Some Nanomedicines

Although the pharmaceutical companies that develop nanomedicines obtain funding from venture capital, capital markets, and partnerships with industries, clinical failure of these products may occur and result in product terminations and business liquidation [147]. Here, we would like to briefly cover the most common causes of nanomedicine clinical trial termination or discontinuation from the market. Nanomedicine toxicity in clinical trials is one of the most common reasons related to clinical failure that usually occurs in phase I trials. For example, the MRX34 nanomedicine, manufactured by Mirna Therapeutics, Austin, TX, USA, was terminated in 2016 in a phase I clinical trial, as one-fifth of patients experienced severe immune-related adverse events [147]. In 2019, due to cumulative neuropathy observed in phase I trials, Merrimack Pharmaceuticals ended the development of MM-310, which was an antibody-directed nanotherapeutic [147]. In addition, the choice of drug carrier is critical in terms of how it works and what the physicochemical properties are. BIND-014, an anticancer treatment for head and neck cancer, failed in a phase II clinical trial (NCT02479178), possibly due to the wrong choice of payload [147].

The right selection of patient is a critical step in successful clinical trials. For instance, involving patients with heterogenous cancer diseases in clinical trials that are established to investigate the safety and efficacy of anticancer treatments could contribute to poor clinical outcomes. Therefore, the development of innovative approaches to control in vivo monitoring of the distribution and transport of nanoparticles will provide a good reference for all researchers in this field [147].

The high-quality production of nanoparticles with reproducibility to meet high manufacturing practice standards is a major challenge in nanomedicine development. Thus, there are some cases of FDA-approved nanomedicines that were discontinued from the market because of manufacturing issues. In 2017, the production of DepoCyt, an injectable nanomedicine used for treating lymphomatous meningitis, was discontinued by Pacira Pharmaceuticals due to unknown technical problems in its manufacturing process [147].

## 8. Future of Nanomedicines

Nanotechnology is already starting to have an impact on the healthcare system. The effect of nanotechnology on healthcare is already being felt, as different nanotechnology ideas have been developed, and several nanotechnology-based medicines are now on the market. Increasing the progress and interest in the field of nanotechnology from the last two decades is predicted for future positive developments in nanomedicines. Therefore, the future of nanomedicines is likely to follow two paths. One path has been already established, where nanomedicines are mainly and still developed for cancer indications; this can be seen in Figure 1, as most nanomedicines currently available in the market are anticancer. Another path is developing nanomedicines to target different diseases other than cancers, which is demonstrated by the recent approvals of Patisiran/ONPATTRO (the first FDA-approved RNAi therapeutic) and VYXEOS (a nanoparticle capable of delivering synergistic ratios of two drugs). More developments along the second path may be required to increase application of nanomedicines to those diseases that cannot be effectively addressed with CDDSs. For instance, nanomedicine in medical diagnosis will enable detection and examination of tissues in more detail: at the cellular, subcellular, and molecular levels, using particular devices [158]. Such a diagnosis would guide the proper treatment, and is called “Personalized Medicine”. Furthermore, to get maximal benefits from the nanoparticle-based strategy for drug delivery, in vitro and in vivo studies need to be further pursued to reach an understanding the behaviors of nanoparticles to accelerate nanomedicines through clinical development, and then provide them to the patients who need them.

Across many parts of the world, nanotechnology draws increasing investment from public authorities and the private sector [2]. In 2016, the global market size of nanomedicine reached USD 138.8 billion, and by 2025, it is projected to hit USD 350.8 billion. This shows the importance of nanotechnology in the field of drug delivery. Reducing undesirable toxicity from nonspecific distribution and improving patient adherence are the main advantages of using nanotechnology to deliver therapeutic agents, with an indirect reduction in the burden on the healthcare system.

## Figures and Tables

**Table 2:** Selected nanomedicines that are in phase III clinical trials.

Type	Proprietary Name	Company	Indication	Active Ingredients	NCT Number	Status	Outcome
Lipid-based Nanoparticles	ThermoDox	Celsion	hepatocellular carcinoma [156]	doxorubicin	NCT00617981	completed	Positive: ThermoDox increased intratumoral concentration of doxorubicin under external hyperthermia induction by 3.7 times compared with ThermoDox without hyperthermia induction
	EndoTAG-1	SynCore Biotechnology	breast cancer	paclitaxel	NCT03002103	ongoing	-
			pancreatic adenocarcinoma [157]	paclitaxel	NCT03126435	ongoing	-
	Allovectin-7^®^	Vical	melanoma	VCL-1005 plasmid	NCT00395070	completed	Nothing has been mentioned
	Tecemotide	Merk KGaA	non-small-cell lung cancer	MUC1 antigen	NCT00409188	completed	Negative: the administration of Tecemotide after chemoradiotherapy compared with placebo showed no significant difference in overall survival for all patients with stage III NSCLC
	MAGE-A3 + AS15	GSK	melanoma	human melanoma-associated antigen A3 protein	NCT00796445	terminated	Negative: MAGE-A3 immunotherapeutic for use in melanoma has been stopped, as it was not efficacious
			non-small-cell lung cancer		NCT00480025	terminated	Negative: MAGE-A3 immunotherapeutic for use in NSCLC has been stopped because it did not increase disease-free survival compared with placebo
	MM-302	Merricmack Pharmaceutical	breast cancer	doxorubicinhydrochloride	NCT02213744	terminated	Negative: MM-302 did not demonstrate benefits over the control
	Taxoprexin	Luitpold Pharmaceuticals	melanoma	paclitaxel	NCT00087776	completed	Nothing has been mentioned
			non-small-cell lung cancer		NCT00243867	completed	Nothing has been mentioned
	Nanocort	Enceladus in collaboration with Sun Pharma Global	rheumatoid arthritis	prednisolone	NCT02534896	terminated	Nothing has been mentioned
Polymer-based Nanoparticles	Nanoplatin	NanoCarrier	advanced solid tumors, lung, biliary, bladder, or pancreatic cancers	cisplatin	NCT02043288	completed	Nothing has been mentioned
	CRLX101	Cerulean	ovarian, renal cell, small cell lung, or rectal cancers	Cyclodextrin-Camptothecin	NCT00163319	completed	Nothing has been mentioned
	NC-6004	NanoCarrier	pancreatic cancer	cisplatin	NCT02043288	completed	Nothing has been mentioned
	NKTR-102	Nektar Therapeutics	breast cancer brain metastases (BCBM)	irinotecan	NCT02915744	completed	Positive: there was a significant improvement in survival of patients with BCBM
	NK-105	NanoCarrier	breast cancer	paclitaxel	NCT01644890	completed	Negative: the progression-free survival, which was the primary outcome measure, was not met
	CT-2103	CTI BioPharma	ovarian, peritoneal, or fallopian tube cancer	paclitaxel	NCT00108745	ongoing	-
			non-small-cell lung cancer		NCT00054210	terminated	Nothing has been mentioned
Inorganic nanoparticles	NBTXR3	Nanobiotix	sarcoma	hafnium-oxide nanoparticle	NCT02379845	ongoing	-
Other	Livatag	Onxeo	hepatocellular carcinoma	doxorubicin	NCT01655693	ongoing	-

## Abbreviations

**Word**	**Abbreviation**
Conventional drug delivery systems	CDDSs
Food and Drug Administration	FDA
European Medicines Agency	EMA
Active pharmaceutical ingredients	APIs
Nanoparticles	NPs
Enhanced permeability and retention	EPR
Gastrointestinal tract	GIT
Reticuloendothelial system	RES
Central nervous system	CNS
Poly(butylcyanoacrylate)	PBCA
Poly(lactic-co-glycolic acid)	PLGA
Poly(lactic acid)	PLA
Federal Food, Drug, and Cosmetic Act	FDCA
Public Health Service Act	PHSA
Nonbiological complex drugs	NBCDs
Pharmacokinetics	PK
Chemistry, manufacturing, and controls	CMC
Nuclear magnetic resonance	NMR
Mass spectrometry	MS
Research and development	R&D
World Health Organization	WHO
Polyethylene glycol	PEG
